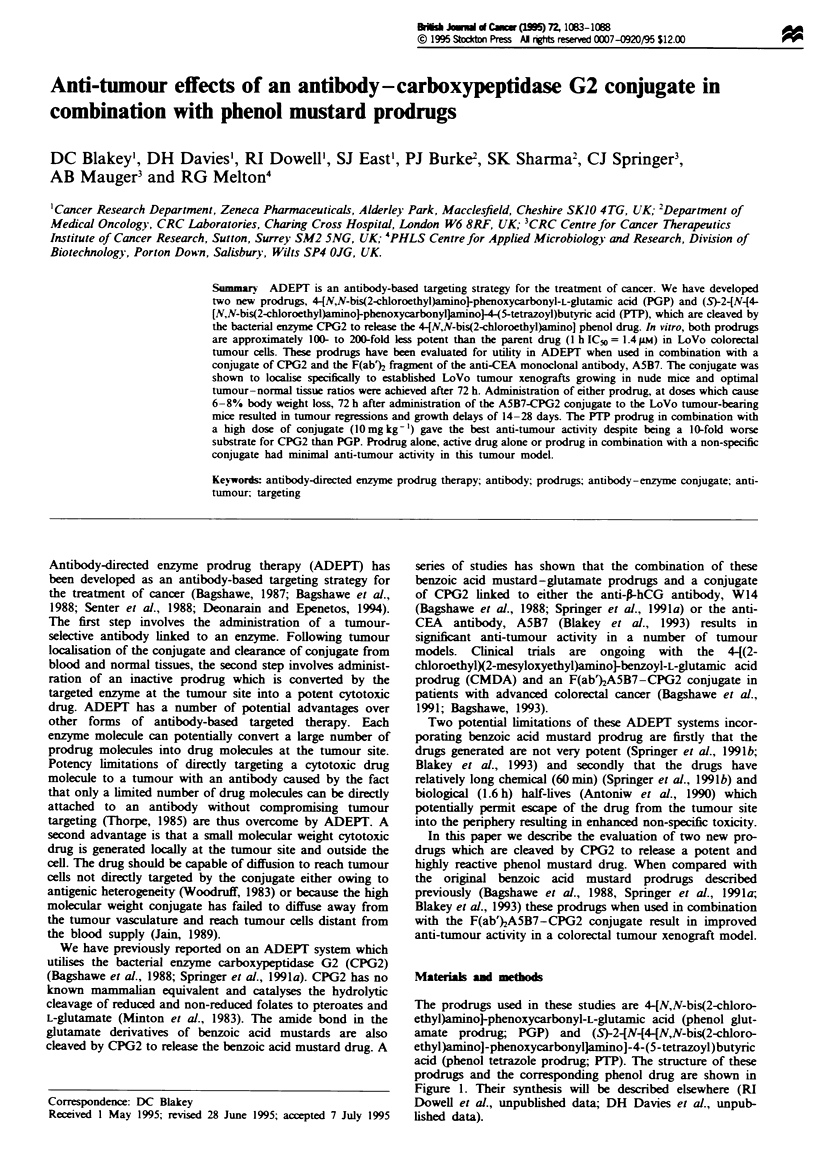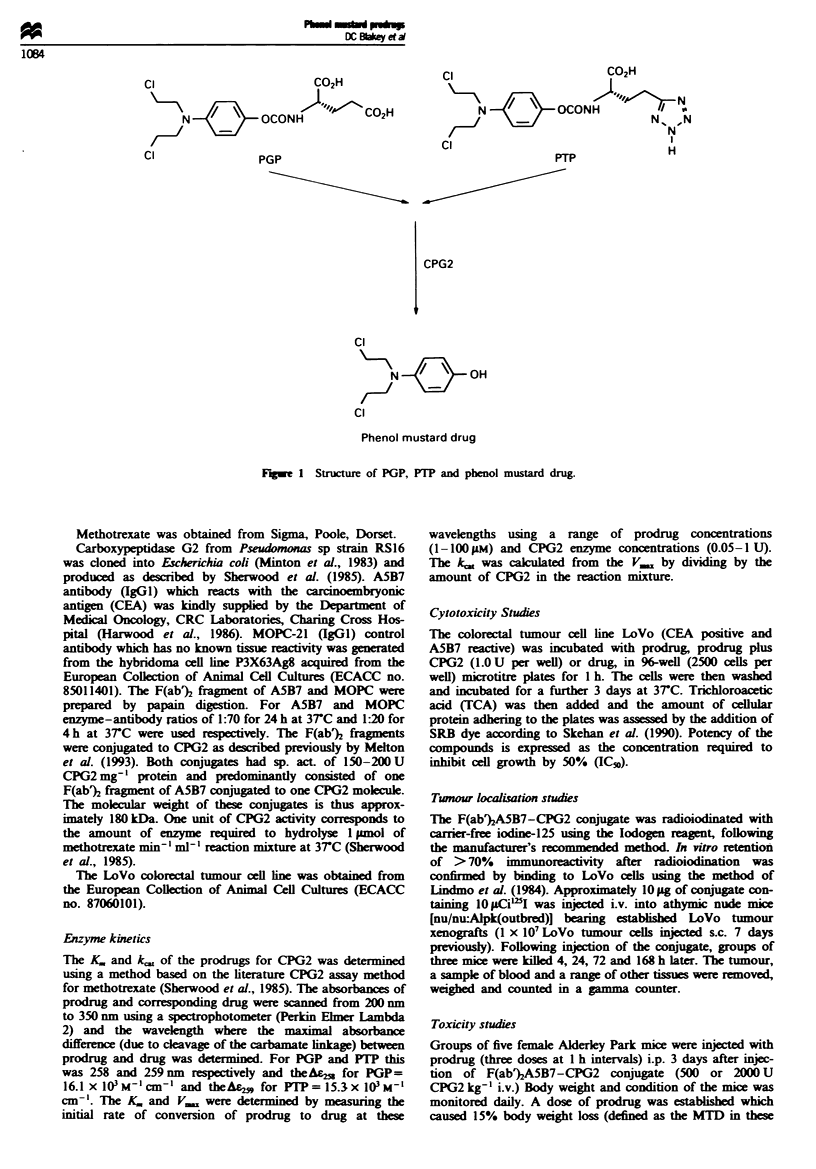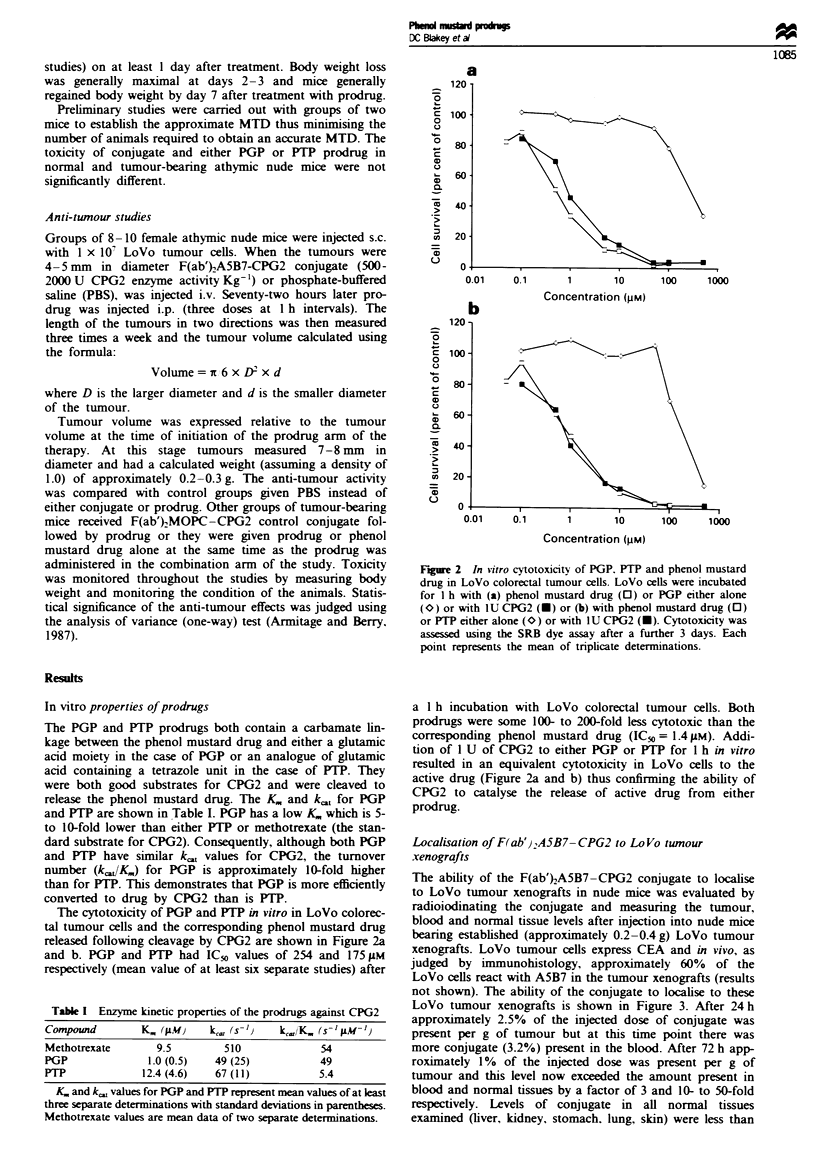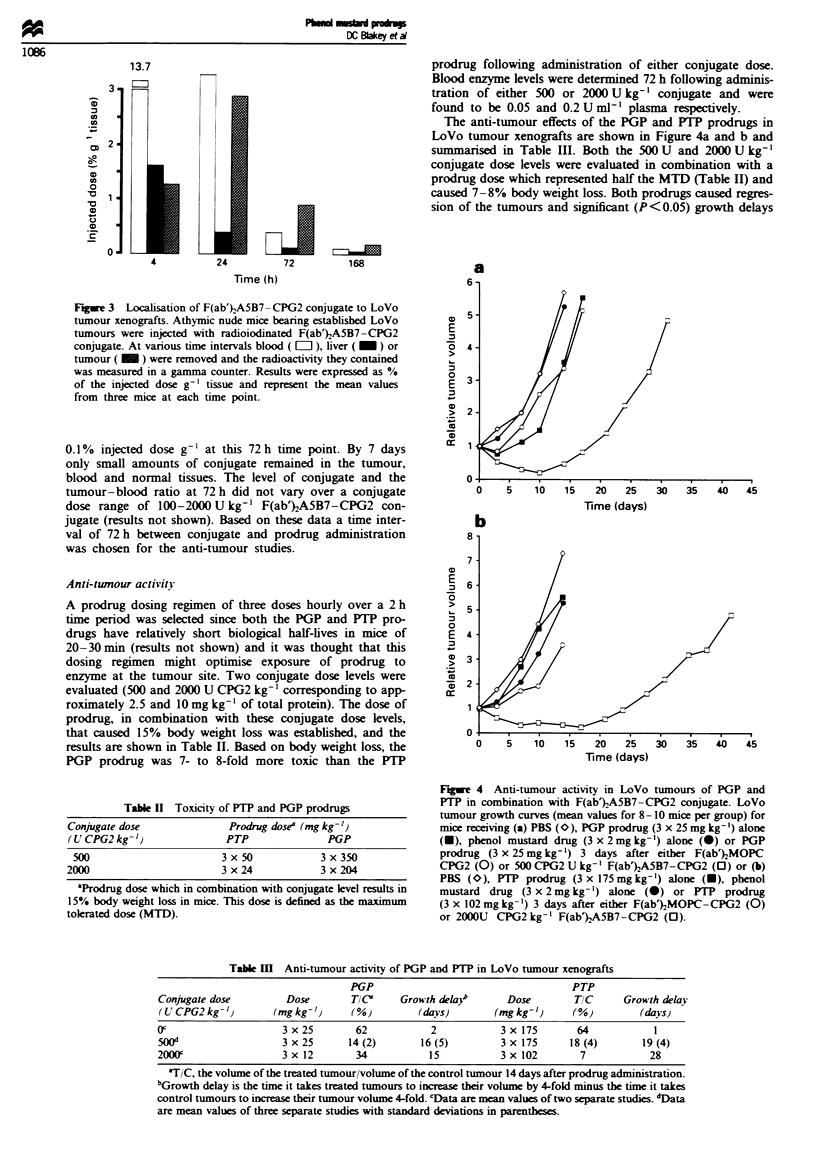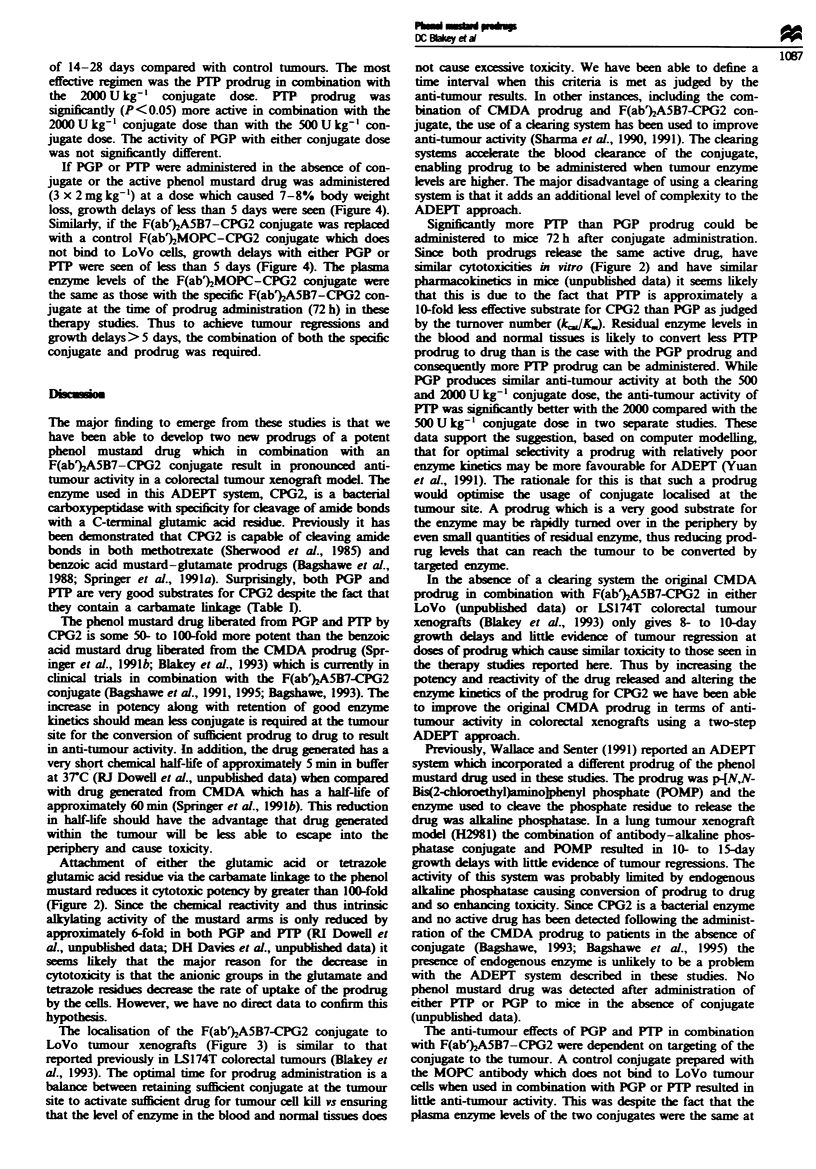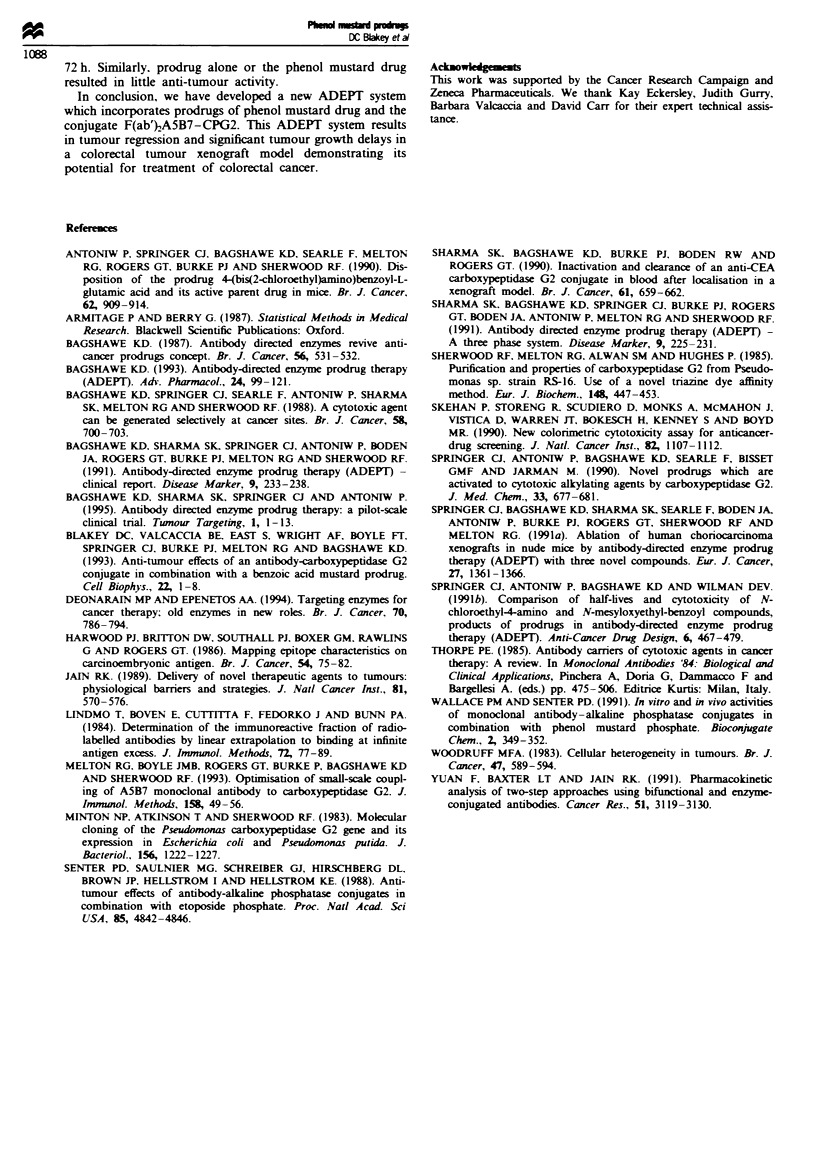# Anti-tumour effects of an antibody-carboxypeptidase G2 conjugate in combination with phenol mustard prodrugs.

**DOI:** 10.1038/bjc.1995.469

**Published:** 1995-11

**Authors:** D. C. Blakey, D. H. Davies, R. I. Dowell, S. J. East, P. J. Burke, S. K. Sharma, C. J. Springer, A. B. Mauger, R. G. Melton

**Affiliations:** Cancer Research Department, Zeneca Pharmaceuticals, Macclesfield, Cheshire, UK.

## Abstract

ADEPT is an antibody-based targeting strategy for the treatment of cancer. We have developed two new prodrugs, 4-[N,N-bis(2-chloroethyl)amino]-phenoxycarbonyl-L- glutamic acid (PGP) and (S)-2-[N-[4-[N,N-bis(2-chloroethyl)amino]- phenoxycarbonyl]amino]-4-(5-tetrazoyl)butyric acid (PTP), which are cleaved by the bacterial enzyme CPG2 to release the 4-[N,N-bis(2-chloroethyl)amino] phenol drug. In vitro, both prodrugs are approximately 100- to 200-fold less potent than the parent drug (1 h IC50 = 1.4 microM) in LoVo colorectal tumour cells. These prodrugs have been evaluated for utility in ADEPT when used in combination with a conjugate of CPG2 and the F(ab')2 fragment of the anti-CEA monoclonal antibody, A5B7. The conjugate was shown to localise specifically to established LoVo tumour xenografts growing in nude mice and optimal tumour-normal tissue ratios were achieved after 72 h. Administration of either prodrug, at doses which cause 6-8% body weight loss, 72 h after administration of the A5B7-CPG2 conjugate to the LoVo tumour-bearing mice resulted in tumour regressions and growth delays of 14-28 days. The PTP prodrug in combination with a high dose of conjugate (10 mg kg-1) gave the best anti-tumour activity despite being a 10-fold worse substrate for CPG2 than PGP. Prodrug alone, active drug alone or prodrug in combination with a non-specific conjugate had minimal anti-tumour activity in this tumour model.


© 1995 Stockton Press All rights reserved 0007-0920/95 $12.00

### Anti-tumour effects of an antibody–carboxypeptidase G2 conjugate in combination with phenol mustard prodrugs

DC Blakey[1], DH Davies[1], RI Dowell[1], SJ East[1], PJ Burke[2], SK Sharma[2], CJ Springer[3], AB Mauger[3] and RG Melton[4]

[1]*Cancer Research Department, Zeneca Pharmaceuticals, Alderley Park, Macclesfield, Cheshire SK10 4TG, UK;* [2]*Department of Medical Oncology, CRC Laboratories, Charing Cross Hospital, London W6 8RF, UK;* [3]*CRC Centre for Cancer Therapeutics Institute of Cancer Research, Sutton, Surrey SM2 5NG, UK;* [4]*PHLS Centre for Applied Microbiology and Research, Division of Biotechnology, Porton Down, Salisbury, Wilts SP4 0JG, UK.*

**Summary** ADEPT is an antibody-based targeting strategy for the treatment of cancer. We have developed two new prodrugs, 4-[*N,N*-bis(2-chloroethyl)amino]-phenoxycarbonyl-L-glutamic acid (PGP) and (*S*)-2-[*N*-[4-[*N,N*-bis(2-chloroethyl)amino]-phenoxycarbonyl]amino]-4-(5-tetrazoyl)butyric acid (PTP), which are cleaved by the bacterial enzyme CPG2 to release the 4-[*N,N*-bis(2-chloroethyl)amino] phenol drug. *In vitro*, both prodrugs are approximately 100- to 200-fold less potent than the parent drug (1 h $IC_{50} = 1.4\ \mu M$) in LoVo colorectal tumour cells. These prodrugs have been evaluated for utility in ADEPT when used in combination with a conjugate of CPG2 and the $F(ab')_2$ fragment of the anti-CEA monoclonal antibody, A5B7. The conjugate was shown to localise specifically to established LoVo tumour xenografts growing in nude mice and optimal tumour–normal tissue ratios were achieved after 72 h. Administration of either prodrug, at doses which cause 6–8% body weight loss, 72 h after administration of the A5B7-CPG2 conjugate to the LoVo tumour-bearing mice resulted in tumour regressions and growth delays of 14–28 days. The PTP prodrug in combination with a high dose of conjugate ($10\ mg\ kg^{-1}$) gave the best anti-tumour activity despite being a 10-fold worse substrate for CPG2 than PGP. Prodrug alone, active drug alone or prodrug in combination with a non-specific conjugate had minimal anti-tumour activity in this tumour model.

**Keywords:** antibody-directed enzyme prodrug therapy; antibody; prodrugs; antibody–enzyme conjugate; anti-tumour; targeting

Antibody-directed enzyme prodrug therapy (ADEPT) has been developed as an antibody-based targeting strategy for the treatment of cancer (Bagshawe, 1987; Bagshawe *et al.*, 1988; Senter *et al.*, 1988; Deonarain and Epenetos, 1994). The first step involves the administration of a tumour-selective antibody linked to an enzyme. Following tumour localisation of the conjugate and clearance of conjugate from blood and normal tissues, the second step involves administration of an inactive prodrug which is converted by the targeted enzyme at the tumour site into a potent cytotoxic drug. ADEPT has a number of potential advantages over other forms of antibody-based targeted therapy. Each enzyme molecule can potentially convert a large number of prodrug molecules into drug molecules at the tumour site. Potency limitations of directly targeting a cytotoxic drug molecule to a tumour with an antibody caused by the fact that only a limited number of drug molecules can be directly attached to an antibody without compromising tumour targeting (Thorpe, 1985) are thus overcome by ADEPT. A second advantage is that a small molecular weight cytotoxic drug is generated locally at the tumour site and outside the cell. The drug should be capable of diffusion to reach tumour cells not directly targeted by the conjugate either owing to antigenic heterogeneity (Woodruff, 1983) or because the high molecular weight conjugate has failed to diffuse away from the tumour vasculature and reach tumour cells distant from the blood supply (Jain, 1989).

We have previously reported on an ADEPT system which utilises the bacterial enzyme carboxypeptidase G2 (CPG2) (Bagshawe *et al.*, 1988; Springer *et al.*, 1991*a*). CPG2 has no known mammalian equivalent and catalyses the hydrolytic cleavage of reduced and non-reduced folates to pteroates and L-glutamate (Minton *et al.*, 1983). The amide bond in the glutamate derivatives of benzoic acid mustards are also cleaved by CPG2 to release the benzoic acid mustard drug. A series of studies has shown that the combination of these benzoic acid mustard–glutamate prodrugs and a conjugate of CPG2 linked to either the anti-β-hCG antibody, W14 (Bagshawe *et al.*, 1988; Springer *et al.*, 1991*a*) or the anti-CEA antibody, A5B7 (Blakey *et al.*, 1993) results in significant anti-tumour activity in a number of tumour models. Clinical trials are ongoing with the 4-[(2-chloroethyl)(2-mesyloxyethyl)amino]-benzoyl-L-glutamic acid prodrug (CMDA) and an $F(ab')_2$A5B7–CPG2 conjugate in patients with advanced colorectal cancer (Bagshawe *et al.*, 1991; Bagshawe, 1993).

Two potential limitations of these ADEPT systems incorporating benzoic acid mustard prodrug are firstly that the drugs generated are not very potent (Springer *et al.*, 1991*b*; Blakey *et al.*, 1993) and secondly that the drugs have relatively long chemical (60 min) (Springer *et al.*, 1991*b*) and biological (1.6 h) half-lives (Antoniw *et al.*, 1990) which potentially permit escape of the drug from the tumour site into the periphery resulting in enhanced non-specific toxicity.

In this paper we describe the evaluation of two new prodrugs which are cleaved by CPG2 to release a potent and highly reactive phenol mustard drug. When compared with the original benzoic acid mustard prodrugs described previously (Bagshawe *et al.*, 1988, Springer *et al.*, 1991*a*; Blakey *et al.*, 1993) these prodrugs when used in combination with the $F(ab')_2$A5B7–CPG2 conjugate result in improved anti-tumour activity in a colorectal tumour xenograft model.

### Materials and methods

The prodrugs used in these studies are 4-[*N,N*-bis(2-chloroethyl)amino]-phenoxycarbonyl-L-glutamic acid (phenol glutamate prodrug; PGP) and (*S*)-2-[*N*-[4-[*N,N*-bis(2-chloroethyl)amino]-phenoxycarbonyl]amino]-4-(5-tetrazoyl)butyric acid (phenol tetrazole prodrug; PTP). The structure of these prodrugs and the corresponding phenol drug are shown in Figure 1. Their synthesis will be described elsewhere (RI Dowell *et al.*, unpublished data; DH Davies *et al.*, unpublished data).

Correspondence: DC Blakey
Received 1 May 1995; revised 28 June 1995; accepted 7 July 1995

**Figure 1** Structure of PGP, PTP and phenol mustard drug.

Methotrexate was obtained from Sigma, Poole, Dorset.

Carboxypeptidase G2 from *Pseudomonas* sp strain RS16 was cloned into *Escherichia coli* (Minton *et al*., 1983) and produced as described by Sherwood *et al*. (1985). A5B7 antibody (IgG1) which reacts with the carcinoembryonic antigen (CEA) was kindly supplied by the Department of Medical Oncology, CRC Laboratories, Charing Cross Hospital (Harwood *et al*., 1986). MOPC-21 (IgG1) control antibody which has no known tissue reactivity was generated from the hybridoma cell line P3X63Ag8 acquired from the European Collection of Animal Cell Cultures (ECACC no. 85011401). The $F(ab')_2$ fragment of A5B7 and MOPC were prepared by papain digestion. For A5B7 and MOPC enzyme–antibody ratios of 1:70 for 24 h at 37°C and 1:20 for 4 h at 37°C were used respectively. The $F(ab')_2$ fragments were conjugated to CPG2 as described previously by Melton *et al*. (1993). Both conjugates had sp. act. of 150–200 U CPG2 $mg^{-1}$ protein and predominantly consisted of one $F(ab')_2$ fragment of A5B7 conjugated to one CPG2 molecule. The molecular weight of these conjugates is thus approximately 180 kDa. One unit of CPG2 activity corresponds to the amount of enzyme required to hydrolyse 1 μmol of methotrexate $min^{-1}$ $ml^{-1}$ reaction mixture at 37°C (Sherwood *et al*., 1985).

The LoVo colorectal tumour cell line was obtained from the European Collection of Animal Cell Cultures (ECACC no. 87060101).

*Enzyme kinetics*

The $K_m$ and $k_{cat}$ of the prodrugs for CPG2 was determined using a method based on the literature CPG2 assay method for methotrexate (Sherwood *et al*., 1985). The absorbances of prodrug and corresponding drug were scanned from 200 nm to 350 nm using a spectrophotometer (Perkin Elmer Lambda 2) and the wavelength where the maximal absorbance difference (due to cleavage of the carbamate linkage) between prodrug and drug was determined. For PGP and PTP this was 258 and 259 nm respectively and the $\Delta\varepsilon_{258}$ for PGP = $16.1 \times 10^3$ $M^{-1}$ $cm^{-1}$ and the $\Delta\varepsilon_{259}$ for PTP = $15.3 \times 10^3$ $M^{-1}$ $cm^{-1}$. The $K_m$ and $V_{max}$ were determined by measuring the initial rate of conversion of prodrug to drug at these wavelengths using a range of prodrug concentrations (1–100 μM) and CPG2 enzyme concentrations (0.05–1 U). The $k_{cat}$ was calculated from the $V_{max}$ by dividing by the amount of CPG2 in the reaction mixture.

*Cytotoxicity Studies*

The colorectal tumour cell line LoVo (CEA positive and A5B7 reactive) was incubated with prodrug, prodrug plus CPG2 (1.0 U per well) or drug, in 96-well (2500 cells per well) microtitre plates for 1 h. The cells were then washed and incubated for a further 3 days at 37°C. Trichloroacetic acid (TCA) was then added and the amount of cellular protein adhering to the plates was assessed by the addition of SRB dye according to Skehan *et al*. (1990). Potency of the compounds is expressed as the concentration required to inhibit cell growth by 50% ($IC_{50}$).

*Tumour localisation studies*

The $F(ab')_2$A5B7–CPG2 conjugate was radioiodinated with carrier-free iodine-125 using the Iodogen reagent, following the manufacturer's recommended method. *In vitro* retention of >70% immunoreactivity after radioiodination was confirmed by binding to LoVo cells using the method of Lindmo *et al*. (1984). Approximately 10 μg of conjugate containing 10 μCi $^{125}I$ was injected i.v. into athymic nude mice [nu/nu:Alpk(outbred)] bearing established LoVo tumour xenografts ($1 \times 10^7$ LoVo tumour cells injected s.c. 7 days previously). Following injection of the conjugate, groups of three mice were killed 4, 24, 72 and 168 h later. The tumour, a sample of blood and a range of other tissues were removed, weighed and counted in a gamma counter.

*Toxicity studies*

Groups of five female Alderley Park mice were injected with prodrug (three doses at 1 h intervals) i.p. 3 days after injection of $F(ab')_2$A5B7–CPG2 conjugate (500 or 2000 U CPG2 $kg^{-1}$ i.v.) Body weight and condition of the mice was monitored daily. A dose of prodrug was established which caused 15% body weight loss (defined as the MTD in these studies) on at least 1 day after treatment. Body weight loss was generally maximal at days 2–3 and mice generally regained body weight by day 7 after treatment with prodrug.

Preliminary studies were carried out with groups of two mice to establish the approximate MTD thus minimising the number of animals required to obtain an accurate MTD. The toxicity of conjugate and either PGP or PTP prodrug in normal and tumour-bearing athymic nude mice were not significantly different.

### *Anti-tumour studies*

Groups of 8–10 female athymic nude mice were injected s.c. with $1 \times 10^7$ LoVo tumour cells. When the tumours were 4–5 mm in diameter $F(ab')_2A5B7$-CPG2 conjugate (500–2000 U CPG2 enzyme activity $Kg^{-1}$) or phosphate-buffered saline (PBS), was injected i.v. Seventy-two hours later prodrug was injected i.p. (three doses at 1 h intervals). The length of the tumours in two directions was then measured three times a week and the tumour volume calculated using the formula:

$$\text{Volume} = \pi\, 6 \times D^2 \times d$$

where $D$ is the larger diameter and $d$ is the smaller diameter of the tumour.

Tumour volume was expressed relative to the tumour volume at the time of initiation of the prodrug arm of the therapy. At this stage tumours measured 7–8 mm in diameter and had a calculated weight (assuming a density of 1.0) of approximately 0.2–0.3 g. The anti-tumour activity was compared with control groups given PBS instead of either conjugate or prodrug. Other groups of tumour-bearing mice received $F(ab')_2MOPC$–CPG2 control conjugate followed by prodrug or they were given prodrug or phenol mustard drug alone at the same time as the prodrug was administered in the combination arm of the study. Toxicity was monitored throughout the studies by measuring body weight and monitoring the condition of the animals. Statistical significance of the anti-tumour effects was judged using the analysis of variance (one-way) test (Armitage and Berry, 1987).

**Figure 2** *In vitro* cytotoxicity of PGP, PTP and phenol mustard drug in LoVo colorectal tumour cells. LoVo cells were incubated for 1 h with (**a**) phenol mustard drug (□) or PGP either alone (◇) or with 1U CPG2 (■) or (**b**) with phenol mustard drug (□) or PTP either alone (◇) or with 1U CPG2 (■). Cytotoxicity was assessed using the SRB dye assay after a further 3 days. Each point represents the mean of triplicate determinations.

### Results

### In vitro *properties of prodrugs*

The PGP and PTP prodrugs both contain a carbamate linkage between the phenol mustard drug and either a glutamic acid moiety in the case of PGP or an analogue of glutamic acid containing a tetrazole unit in the case of PTP. They were both good substrates for CPG2 and were cleaved to release the phenol mustard drug. The $K_m$ and $k_{cat}$ for PGP and PTP are shown in Table I. PGP has a low $K_m$ which is 5- to 10-fold lower than either PTP or methotrexate (the standard substrate for CPG2). Consequently, although both PGP and PTP have similar $k_{cat}$ values for CPG2, the turnover number ($k_{cat}/K_m$) for PGP is approximately 10-fold higher than for PTP. This demonstrates that PGP is more efficiently converted to drug by CPG2 than is PTP.

The cytotoxicity of PGP and PTP *in vitro* in LoVo colorectal tumour cells and the corresponding phenol mustard drug released following cleavage by CPG2 are shown in Figure 2a and b. PGP and PTP had $IC_{50}$ values of 254 and 175 μM respectively (mean value of at least six separate studies) after a 1 h incubation with LoVo colorectal tumour cells. Both prodrugs were some 100- to 200-fold less cytotoxic than the corresponding phenol mustard drug ($IC_{50}$ = 1.4 μM). Addition of 1 U of CPG2 to either PGP or PTP for 1 h *in vitro* resulted in an equivalent cytotoxicity in LoVo cells to the active drug (Figure 2a and b) thus confirming the ability of CPG2 to catalyse the release of active drug from either prodrug.

**Table I** Enzyme kinetic properties of the prodrugs against CPG2

| *Compound* | $K_m$ (μM) | $k_{cat}$ ($s^{-1}$) | $k_{cat}/K_m$ ($s^{-1}$ μ$M^{-1}$) |
|---|---|---|---|
| Methotrexate | 9.5 | 510 | 54 |
| PGP | 1.0 (0.5) | 49 (25) | 49 |
| PTP | 12.4 (4.6) | 67 (11) | 5.4 |

$K_m$ and $k_{cat}$ values for PGP and PTP represent mean values of at least three separate determinations with standard deviations in parentheses. Methotrexate values are mean data of two separate determinations.

### *Localisation of $F(ab')_2A5B7$–CPG2 to LoVo tumour xenografts*

The ability of the $F(ab')_2A5B7$–CPG2 conjugate to localise to LoVo tumour xenografts in nude mice was evaluated by radioiodinating the conjugate and measuring the tumour, blood and normal tissue levels after injection into nude mice bearing established (approximately 0.2–0.4 g) LoVo tumour xenografts. LoVo tumour cells express CEA and *in vivo*, as judged by immunohistology, approximately 60% of the LoVo cells react with A5B7 in the tumour xenografts (results not shown). The ability of the conjugate to localise to these LoVo tumour xenografts is shown in Figure 3. After 24 h approximately 2.5% of the injected dose of conjugate was present per g of tumour but at this time point there was more conjugate (3.2%) present in the blood. After 72 h approximately 1% of the injected dose was present per g of tumour and this level now exceeded the amount present in blood and normal tissues by a factor of 3 and 10- to 50-fold respectively. Levels of conjugate in all normal tissues examined (liver, kidney, stomach, lung, skin) were less than

**Figure 3** Localisation of $F(ab')_2$A5B7-CPG2 conjugate to LoVo tumour xenografts. Athymic nude mice bearing established LoVo tumours were injected with radioiodinated $F(ab')_2$A5B7-CPG2 conjugate. At various time intervals blood ( □ ), liver ( ■ ) or tumour ( ▩ ) were removed and the radioactivity they contained was measured in a gamma counter. Results were expressed as % of the injected dose g$^{-1}$ tissue and represent the mean values from three mice at each time point.

0.1% injected dose g$^{-1}$ at this 72 h time point. By 7 days only small amounts of conjugate remained in the tumour, blood and normal tissues. The level of conjugate and the tumour-blood ratio at 72 h did not vary over a conjugate dose range of 100-2000 U kg$^{-1}$ $F(ab')_2$A5B7-CPG2 conjugate (results not shown). Based on these data a time interval of 72 h between conjugate and prodrug administration was chosen for the anti-tumour studies.

### *Anti-tumour activity*

A prodrug dosing regimen of three doses hourly over a 2 h time period was selected since both the PGP and PTP prodrugs have relatively short biological half-lives in mice of 20-30 min (results not shown) and it was thought that this dosing regimen might optimise exposure of prodrug to enzyme at the tumour site. Two conjugate dose levels were evaluated (500 and 2000 U CPG2 kg$^{-1}$ corresponding to approximately 2.5 and 10 mg kg$^{-1}$ of total protein). The dose of prodrug, in combination with these conjugate dose levels, that caused 15% body weight loss was established, and the results are shown in Table II. Based on body weight loss, the PGP prodrug was 7- to 8-fold more toxic than the PTP prodrug following administration of either conjugate dose. Blood enzyme levels were determined 72 h following administration of either 500 or 2000 U kg$^{-1}$ conjugate and were found to be 0.05 and 0.2 U ml$^{-1}$ plasma respectively.

The anti-tumour effects of the PGP and PTP prodrugs in LoVo tumour xenografts are shown in Figure 4a and b and summarised in Table III. Both the 500 U and 2000 U kg$^{-1}$ conjugate dose levels were evaluated in combination with a prodrug dose which represented half the MTD (Table II) and caused 7-8% body weight loss. Both prodrugs caused regression of the tumours and significant ($P < 0.05$) growth delays

**Table II** Toxicity of PTP and PGP prodrugs

| *Conjugate dose (U CPG2 kg$^{-1}$)* | *Prodrug dose[a] (mg kg$^{-1}$)* PTP | PGP |
|---|---|---|
| 500 | 3 x 50 | 3 x 350 |
| 2000 | 3 x 24 | 3 x 204 |

[a]Prodrug dose which in combination with conjugate level results in 15% body weight loss in mice. This dose is defined as the maximum tolerated dose (MTD).

**Figure 4** Anti-tumour activity in LoVo tumours of PGP and PTP in combination with $F(ab')_2$A5B7-CPG2 conjugate. LoVo tumour growth curves (mean values for 8-10 mice per group) for mice receiving (**a**) PBS (◇), PGP prodrug (3 x 25 mg kg$^{-1}$) alone (■), phenol mustard drug (3 x 2 mg kg$^{-1}$) alone (●) or PGP prodrug (3 x 25 mg kg$^{-1}$) 3 days after either $F(ab')_2$MOPC CPG2 (○) or 500 CPG2 U kg$^{-1}$ $F(ab')_2$A5B7-CPG2 (□) or (**b**) PBS (◇), PTP prodrug (3 x 175 mg kg$^{-1}$) alone (■), phenol mustard drug (3 x 2 mg kg$^{-1}$) alone (●) or PTP prodrug (3 x 102 mg kg$^{-1}$) 3 days after either $F(ab')_2$MOPC-CPG2 (○) or 2000U CPG2 kg$^{-1}$ $F(ab')_2$A5B7-CPG2 (□).

**Table III** Anti-tumour activity of PGP and PTP in LoVo tumour xenografts

| *Conjugate dose (U CPG2 kg$^{-1}$)* | *Dose (mg kg$^{-1}$)* | *PGP T/C[a] (%)* | *Growth delay[b] (days)* | *Dose (mg kg$^{-1}$)* | *PTP T/C (%)* | *Growth delay (days)* |
|---|---|---|---|---|---|---|
| 0[c] | 3 x 25 | 62 | 2 | 3 x 175 | 64 | 1 |
| 500[d] | 3 x 25 | 14 (2) | 16 (5) | 3 x 175 | 18 (4) | 19 (4) |
| 2000[c] | 3 x 12 | 34 | 15 | 3 x 102 | 7 | 28 |

[a]T/C, the volume of the treated tumour/volume of the control tumour 14 days after prodrug administration. [b]Growth delay is the time it takes treated tumours to increase their volume by 4-fold minus the time it takes control tumours to increase their tumour volume 4-fold. [c]Data are mean values of two separate studies. [d]Data are mean values of three separate studies with standard deviations in parentheses.

of 14–28 days compared with control tumours. The most effective regimen was the PTP prodrug in combination with the 2000 U kg$^{-1}$ conjugate dose. PTP prodrug was significantly ($P<0.05$) more active in combination with the 2000 U kg$^{-1}$ conjugate dose than with the 500 U kg$^{-1}$ conjugate dose. The activity of PGP with either conjugate dose was not significantly different.

If PGP or PTP were administered in the absence of conjugate or the active phenol mustard drug was administered (3 × 2 mg kg$^{-1}$) at a dose which caused 7–8% body weight loss, growth delays of less than 5 days were seen (Figure 4). Similarly, if the F(ab')$_2$A5B7–CPG2 conjugate was replaced with a control F(ab')$_2$MOPC–CPG2 conjugate which does not bind to LoVo cells, growth delays with either PGP or PTP were seen of less than 5 days (Figure 4). The plasma enzyme levels of the F(ab')$_2$MOPC–CPG2 conjugate were the same as those with the specific F(ab')$_2$A5B7–CPG2 conjugate at the time of prodrug administration (72 h) in these therapy studies. Thus to achieve tumour regressions and growth delays > 5 days, the combination of both the specific conjugate and prodrug was required.

### Discussion

The major finding to emerge from these studies is that we have been able to develop two new prodrugs of a potent phenol mustard drug which in combination with an F(ab')$_2$A5B7–CPG2 conjugate result in pronounced anti-tumour activity in a colorectal tumour xenograft model. The enzyme used in this ADEPT system, CPG2, is a bacterial carboxypeptidase with specificity for cleavage of amide bonds with a C-terminal glutamic acid residue. Previously it has been demonstrated that CPG2 is capable of cleaving amide bonds in both methotrexate (Sherwood *et al.*, 1985) and benzoic acid mustard–glutamate prodrugs (Bagshawe *et al.*, 1988; Springer *et al.*, 1991*a*). Surprisingly, both PGP and PTP are very good substrates for CPG2 despite the fact that they contain a carbamate linkage (Table I).

The phenol mustard drug liberated from PGP and PTP by CPG2 is some 50- to 100-fold more potent than the benzoic acid mustard drug liberated from the CMDA prodrug (Springer *et al.*, 1991*b*; Blakey *et al.*, 1993) which is currently in clinical trials in combination with the F(ab')$_2$A5B7-CPG2 conjugate (Bagshawe *et al.*, 1991, 1995; Bagshawe, 1993). The increase in potency along with retention of good enzyme kinetics should mean less conjugate is required at the tumour site for the conversion of sufficient prodrug to drug to result in anti-tumour activity. In addition, the drug generated has a very short chemical half-life of approximately 5 min in buffer at 37°C (RJ Dowell *et al.*, unpublished data) when compared with drug generated from CMDA which has a half-life of approximately 60 min (Springer *et al.*, 1991*b*). This reduction in half-life should have the advantage that drug generated within the tumour will be less able to escape into the periphery and cause toxicity.

Attachment of either the glutamic acid or tetrazole glutamic acid residue via the carbamate linkage to the phenol mustard reduces it cytotoxic potency by greater than 100-fold (Figure 2). Since the chemical reactivity and thus intrinsic alkylating activity of the mustard arms is only reduced by approximately 6-fold in both PGP and PTP (RI Dowell *et al.*, unpublished data; DH Davies *et al.*, unpublished data) it seems likely that the major reason for the decrease in cytotoxicity is that the anionic groups in the glutamate and tetrazole residues decrease the rate of uptake of the prodrug by the cells. However, we have no direct data to confirm this hypothesis.

The localisation of the F(ab')$_2$A5B7-CPG2 conjugate to LoVo tumour xenografts (Figure 3) is similar to that reported previously in LS174T colorectal tumours (Blakey *et al.*, 1993). The optimal time for prodrug administration is a balance between retaining sufficient conjugate at the tumour site to activate sufficient drug for tumour cell kill *vs* ensuring that the level of enzyme in the blood and normal tissues does not cause excessive toxicity. We have been able to define a time interval when this criteria is met as judged by the anti-tumour results. In other instances, including the combination of CMDA prodrug and F(ab')$_2$A5B7-CPG2 conjugate, the use of a clearing system has been used to improve anti-tumour activity (Sharma *et al.*, 1990, 1991). The clearing systems accelerate the blood clearance of the conjugate, enabling prodrug to be administered when tumour enzyme levels are higher. The major disadvantage of using a clearing system is that it adds an additional level of complexity to the ADEPT approach.

Significantly more PTP than PGP prodrug could be administered to mice 72 h after conjugate administration. Since both prodrugs release the same active drug, have similar cytotoxicities *in vitro* (Figure 2) and have similar pharmacokinetics in mice (unpublished data) it seems likely that this is due to the fact that PTP is approximately a 10-fold less effective substrate for CPG2 than PGP as judged by the turnover number ($k_{cat}/K_m$). Residual enzyme levels in the blood and normal tissues is likely to convert less PTP prodrug to drug than is the case with the PGP prodrug and consequently more PTP prodrug can be administered. While PGP produces similar anti-tumour activity at both the 500 and 2000 U kg$^{-1}$ conjugate dose, the anti-tumour activity of PTP was significantly better with the 2000 compared with the 500 U kg$^{-1}$ conjugate dose in two separate studies. These data support the suggestion, based on computer modelling, that for optimal selectivity a prodrug with relatively poor enzyme kinetics may be more favourable for ADEPT (Yuan *et al.*, 1991). The rationale for this is that such a prodrug would optimise the usage of conjugate localised at the tumour site. A prodrug which is a very good substrate for the enzyme may be rapidly turned over in the periphery by even small quantities of residual enzyme, thus reducing prodrug levels that can reach the tumour to be converted by targeted enzyme.

In the absence of a clearing system the original CMDA prodrug in combination with F(ab')$_2$A5B7-CPG2 in either LoVo (unpublished data) or LS174T colorectal tumour xenografts (Blakey *et al.*, 1993) only gives 8- to 10-day growth delays and little evidence of tumour regression at doses of prodrug which cause similar toxicity to those seen in the therapy studies reported here. Thus by increasing the potency and reactivity of the drug released and altering the enzyme kinetics of the prodrug for CPG2 we have been able to improve the original CMDA prodrug in terms of anti-tumour activity in colorectal xenografts using a two-step ADEPT approach.

Previously, Wallace and Senter (1991) reported an ADEPT system which incorporated a different prodrug of the phenol mustard drug used in these studies. The prodrug was p-[*N*,*N*-Bis(2-chloroethyl)amino]phenyl phosphate (POMP) and the enzyme used to cleave the phosphate residue to release the drug was alkaline phosphatase. In a lung tumour xenograft model (H2981) the combination of antibody–alkaline phosphatase conjugate and POMP resulted in 10- to 15-day growth delays with little evidence of tumour regressions. The activity of this system was probably limited by endogenous alkaline phosphatase causing conversion of prodrug to drug and so enhancing toxicity. Since CPG2 is a bacterial enzyme and no active drug has been detected following the administration of the CMDA prodrug to patients in the absence of conjugate (Bagshawe, 1993; Bagshawe *et al.*, 1995) the presence of endogenous enzyme is unlikely to be a problem with the ADEPT system described in these studies. No phenol mustard drug was detected after administration of either PTP or PGP to mice in the absence of conjugate (unpublished data).

The anti-tumour effects of PGP and PTP in combination with F(ab')$_2$A5B7–CPG2 were dependent on targeting of the conjugate to the tumour. A control conjugate prepared with the MOPC antibody which does not bind to LoVo tumour cells when used in combination with PGP or PTP resulted in little anti-tumour activity. This was despite the fact that the plasma enzyme levels of the two conjugates were the same at

72 h. Similarly, prodrug alone or the phenol mustard drug resulted in little anti-tumour activity.

In conclusion, we have developed a new ADEPT system which incorporates prodrugs of phenol mustard drug and the conjugate $F(ab')_2A5B7$-CPG2. This ADEPT system results in tumour regression and significant tumour growth delays in a colorectal tumour xenograft model demonstrating its potential for treatment of colorectal cancer.

**Acknowledgements**

This work was supported by the Cancer Research Campaign and Zeneca Pharmaceuticals. We thank Kay Eckersley, Judith Gurry, Barbara Valcaccia and David Carr for their expert technical assistance.

**References**

ANTONIW P, SPRINGER CJ, BAGSHAWE KD, SEARLE F, MELTON RG, ROGERS GT, BURKE PJ AND SHERWOOD RF. (1990). Disposition of the prodrug 4-(bis(2-chloroethyl)amino)benzoyl-L-glutamic acid and its active parent drug in mice. *Br. J. Cancer*, **62**, 909-914.

ARMITAGE P AND BERRY G. (1987). *Statistical Methods in Medical Research*. Blackwell Scientific Publications: Oxford.

BAGSHAWE KD. (1987). Antibody directed enzymes revive anti-cancer prodrugs concept. *Br. J. Cancer*, **56**, 531-532.

BAGSHAWE KD. (1993). Antibody-directed enzyme prodrug therapy (ADEPT). *Adv. Pharmacol.*, **24**, 99-121.

BAGSHAWE KD, SPRINGER CJ, SEARLE F, ANTONIW P, SHARMA SK, MELTON RG AND SHERWOOD RF. (1988). A cytotoxic agent can be generated selectively at cancer sites. *Br. J. Cancer*, **58**, 700-703.

BAGSHAWE KD, SHARMA SK, SPRINGER CJ, ANTONIW P, BODEN JA, ROGERS GT, BURKE PJ, MELTON RG AND SHERWOOD RF. (1991). Antibody-directed enzyme prodrug therapy (ADEPT) - clinical report. *Disease Marker*, **9**, 233-238.

BAGSHAWE KD, SHARMA SK, SPRINGER CJ AND ANTONIW P. (1995). Antibody directed enzyme prodrug therapy: a pilot-scale clinical trial. *Tumour Targeting*, **1**, 1-13.

BLAKEY DC, VALCACCIA BE, EAST S, WRIGHT AF, BOYLE FT, SPRINGER CJ, BURKE PJ, MELTON RG AND BAGSHAWE KD. (1993). Anti-tumour effects of an antibody-carboxypeptidase G2 conjugate in combination with a benzoic acid mustard prodrug. *Cell Biophys.*, **22**, 1-8.

DEONARAIN MP AND EPENETOS AA. (1994). Targeting enzymes for cancer therapy; old enzymes in new roles. *Br. J. Cancer*, **70**, 786-794.

HARWOOD PJ, BRITTON DW, SOUTHALL PJ, BOXER GM, RAWLINS G AND ROGERS GT. (1986). Mapping epitope characteristics on carcinoembryonic antigen. *Br. J. Cancer*, **54**, 75-82.

JAIN RK. (1989). Delivery of novel therapeutic agents to tumours: physiological barriers and strategies. *J. Natl Cancer Inst.*, **81**, 570-576.

LINDMO T, BOVEN E, CUTTITTA F, FEDORKO J AND BUNN PA. (1984). Determination of the immunoreactive fraction of radiolabelled antibodies by linear extrapolation to binding at infinite antigen excess. *J. Immunol. Methods*, **72**, 77-89.

MELTON RG, BOYLE JMB, ROGERS GT, BURKE P, BAGSHAWE KD AND SHERWOOD RF. (1993). Optimisation of small-scale coupling of A5B7 monoclonal antibody to carboxypeptidase G2. *J. Immunol. Methods*, **158**, 49-56.

MINTON NP, ATKINSON T AND SHERWOOD RF. (1983). Molecular cloning of the *Pseudomonas* carboxypeptidase G2 gene and its expression in *Escherichia coli* and *Pseudomonas putida*. *J. Bacteriol.*, **156**, 1222-1227.

SENTER PD, SAULNIER MG, SCHREIBER GJ, HIRSCHBERG DL, BROWN JP, HELLSTROM I AND HELLSTROM KE. (1988). Anti-tumour effects of antibody-alkaline phosphatase conjugates in combination with etoposide phosphate. *Proc. Natl Acad. Sci USA*, **85**, 4842-4846.

SHARMA SK, BAGSHAWE KD, BURKE PJ, BODEN RW AND ROGERS GT. (1990). Inactivation and clearance of an anti-CEA carboxypeptidase G2 conjugate in blood after localisation in a xenograft model. *Br. J. Cancer*, **61**, 659-662.

SHARMA SK, BAGSHAWE KD, SPRINGER CJ, BURKE PJ, ROGERS GT, BODEN JA, ANTONIW P, MELTON RG AND SHERWOOD RF. (1991). Antibody directed enzyme prodrug therapy (ADEPT) - A three phase system. *Disease Marker*, **9**, 225-231.

SHERWOOD RF, MELTON RG, ALWAN SM AND HUGHES P. (1985). Purification and properties of carboxypeptidase G2 from Pseudomonas sp. strain RS-16. Use of a novel triazine dye affinity method. *Eur. J. Biochem.*, **148**, 447-453.

SKEHAN P, STORENG R, SCUDIERO D, MONKS A, MCMAHON J, VISTICA D, WARREN JT, BOKESCH H, KENNEY S AND BOYD MR. (1990). New colorimetric cytotoxicity assay for anticancer-drug screening. *J. Natl. Cancer Inst.*, **82**, 1107-1112.

SPRINGER CJ, ANTONIW P, BAGSHAWE KD, SEARLE F, BISSET GMF AND JARMAN M. (1990). Novel prodrugs which are activated to cytotoxic alkylating agents by carboxypeptidase G2. *J. Med. Chem.*, **33**, 677-681.

SPRINGER CJ, BAGSHAWE KD, SHARMA SK, SEARLE F, BODEN JA, ANTONIW P, BURKE PJ, ROGERS GT, SHERWOOD RF AND MELTON RG. (1991*a*). Ablation of human choriocarcinoma xenografts in nude mice by antibody-directed enzyme prodrug therapy (ADEPT) with three novel compounds. *Eur. J. Cancer*, **27**, 1361-1366.

SPRINGER CJ, ANTONIW P, BAGSHAWE KD AND WILMAN DEV. (1991*b*). Comparison of half-lives and cytotoxicity of *N*-chloroethyl-4-amino and *N*-mesyloxyethyl-benzoyl compounds, products of prodrugs in antibody-directed enzyme prodrug therapy (ADEPT). *Anti-Cancer Drug Design*, **6**, 467-479.

THORPE PE. (1985). Antibody carriers of cytotoxic agents in cancer therapy: A review. In *Monoclonal Antibodies '84: Biological and Clinical Applications*, Pinchera A, Doria G, Dammacco F and Bargellesi A. (eds.) pp. 475-506. Editrice Kurtis: Milan, Italy.

WALLACE PM AND SENTER PD. (1991). *In vitro* and *in vivo* activities of monoclonal antibody-alkaline phosphatase conjugates in combination with phenol mustard phosphate. *Bioconjugate Chem.*, **2**, 349-352.

WOODRUFF MFA. (1983). Cellular heterogeneity in tumours. *Br. J. Cancer*, **47**, 589-594.

YUAN F, BAXTER LT AND JAIN RK. (1991). Pharmacokinetic analysis of two-step approaches using bifunctional and enzyme-conjugated antibodies. *Cancer Res.*, **51**, 3119-3130.